# The Anxiolytic Drug Buspirone Prevents Rotenone-Induced Toxicity in a Mouse Model of Parkinson’s Disease

**DOI:** 10.3390/ijms23031845

**Published:** 2022-02-06

**Authors:** Sarah Thomas Broome, Alessandro Castorina

**Affiliations:** Laboratory of Cellular and Molecular Neuroscience (LCMN), School of Life Sciences, Faculty of Science, University of Technology Sydney, Sydney, NSW 2007, Australia; sarah.j.thomasbroome@student.uts.edu.au

**Keywords:** Parkinson’s disease, neurodegeneration, rotenone, neuroinflammation, dopamine, dopamine-D3-receptor, buspirone

## Abstract

A pharmacological and genetic blockade of the dopamine D3 receptor (D3R) has shown to be neuroprotective in models of Parkinson’s disease (PD). The anxiolytic drug buspirone, a serotonin receptor 1A agonist, also functions as a potent D3R antagonist. To test if buspirone elicited neuroprotective activities, C57BL/6 mice were subjected to rotenone treatment (10mg/kg i.p for 21 days) to induce PD-like pathology and were co-treated with increasing dosages of buspirone (1, 3, or 10 mg/kg i.p.) to determine if the drug could prevent rotenone-induced damage to the central nervous system (CNS). We found that high dosages of buspirone prevented the behavioural deficits caused by rotenone in the open field test. Molecular and histological analyses confirmed that 10 mg/kg of buspirone prevented the degeneration of TH-positive neurons. Buspirone attenuated the induction of interleukin-1β and interleukin-6 expression by rotenone, and this was paralleled by the upregulation of arginase-1, brain-derived neurotrophic factor (BDNF), and activity-dependent neuroprotective protein (ADNP) in the midbrain, striatum, prefrontal cortex, amygdala, and hippocampus. Buspirone treatment also improved mitochondrial function and antioxidant activities. Lastly, the drug prevented the disruptions in the expression of two neuroprotective peptides, pituitary adenylate cyclase-activating polypeptide (PACAP) and vasoactive intestinal peptide (VIP). These results pinpoint the neuroprotective efficacy of buspirone against rotenone toxicity, suggesting its potential use as a therapeutic agent in neurodegenerative and neuroinflammatory diseases, such as PD.

## 1. Introduction

Parkinson’s disease (PD) is the most common neurodegenerative movement disorder, characterised by the progressive loss of dopaminergic neurons in the *substantia nigra pars compacta* (SNpc) and resulting in a deficit of dopamine (DA) in the *striatum* [1]. PD is a multi-factorial disease that arises from a complex interplay between several environmental factors, a genetic predisposition, and defective cellular processes [2,3]. Many attempts were made in rodents to reproduce some of the pathological domains of PD. For example, the environmental toxin, rotenone, is a popular PD-mimetic due to its ability to reproduce the major clinical and behavioural features of PD in rodents, including motor deficits, dopaminergic degeneration, mitochondrial impairment, and neuroinflammation [4,5]. 

Neuroinflammation describes the local immune response within the central nervous system (CNS), and is predominantly driven by resident microglia [6,7]. It is a major contributor to PD pathology and is consistently linked to disease progression and clinical severity [8]. Furthermore, it was shown that neuroinflammation alone is sufficient to promote the death of dopaminergic neurons, as shown in a study involving an injection of the inflammatory mimetic, LPS, into the rodent brain, which resulted in increased levels of inflammatory mediators prior to the loss of dopaminergic neurons [8]. Adding complexity to PD pathogenesis is the discovery that DA itself can control inflammation [9]. 

The discovery of functional dopamine receptors expressed on the surface of multiple immune cell subtypes, including microglia [10], suggests that DA itself can modulate at least certain immune responses [9]. This is clearly seen in the ability of the dopamine D3 receptor (D3R) to promote inflammation. It was shown that D3R-signalling promotes disease progression by favouring neuroinflammation and promoting the pathogenic CD4+ T cell response associated with PD [11,12]. Additionally, microglial activation is repressed in D3R-deficient mice [11]. Most importantly, compounds able to block D3R reduced CNS inflammation and, consequently, slowed the progression of PD [11,13].

Computational and neuroimaging studies have revealed that the anxiolytic drug buspirone, a partial 5-hydroxytryptamine receptor (5-HT1Ar) agonist, has a strong “off-target” function as a D3R antagonist [14,15,16]. In recent work, we demonstrated that either the genetic deletion of D3R or buspirone treatment reliably attenuated LPS-triggered inflammation in BV2 microglia [17]. It is well accepted that blocking microglial-induced inflammation reduces neurodegeneration and slows disease progression [18,19]. 

The emerging evidence extending the pharmacological properties of buspirone, together with the neuroprotective effects seen in response to D3R blockade, prompted us to investigate if buspirone can protect from dopaminergic degeneration by attenuating neuroinflammation in a rotenone mouse model of PD. We focused on the ability of buspirone to protect against rotenone-induced behavioural deficits, dopaminergic degeneration, mitochondrial dysfunction, and inflammation. To the best of our knowledge, this is the first study to characterise the neurochemical changes triggered by buspirone as an anti-inflammatory agent in several CNS structures. Our results indicate that, at high dosages, buspirone is able to prevent rotenone-induced deficits in locomotor and exploratory behaviour, protect dopaminergic neurons from degeneration, and reduce the expression of inflammatory mediators whilst heightening the expression of neurotrophic factors and protective neuropeptides in several CNS regions. Collectively, these findings support the idea that buspirone protects the CNS against rotenone intoxication in a mouse model of PD. 

## 2. Results

### 2.1. Buspirone Prevents Rotenone-Induced Deficits in Locomotor and Exploratory Behaviour

**Figure 1:**
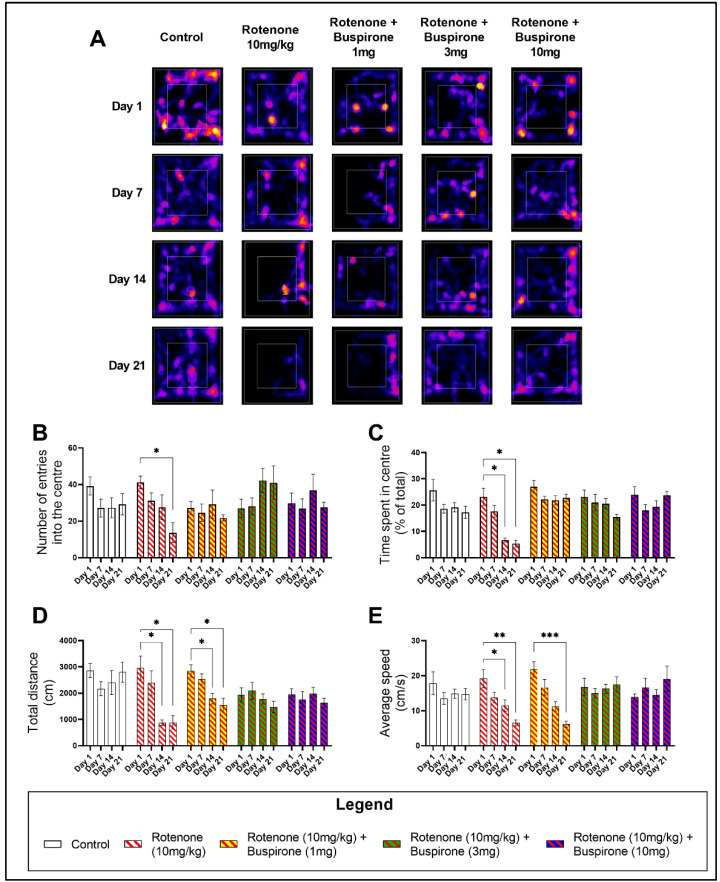
Buspirone prevents rotenone-induced locomotor and exploratory behavioural deficits. The open field (OF) test was used to assess the locomotor and exploratory behaviours of mice on a weekly basis for a total of 21 days. Representative heat maps generated from MouBeAt Software illustrate the locomotor pattern of mice during 5 min spent in the OF (**A**). Exploratory behaviour was determined by measuring the number of entries (**B**) and the total time (**C**) each mouse spent in the centre quadrant. Locomotor activity was assessed by comparing the total distance travelled (**D**) and average speed (**E**) of mice. Comparisons were made within the same treatment group compared to baseline measurements. Data shown represents 8–12 mice per group. * *p* < 0.05, ** *p* < 0.01, or *** *p* < 0.001 as determined by a one-way ANOVA followed by Dunnett’s post-hoc test.

Buspirone is clinically used as an anxiolytic and has been previously utilised as a positive control in the assessment of the exploratory behaviour of C57BL/6 mice in the open field (OF) [20] (Supplementary Figure S1). Rotenone (10 mg/kg) induced significant deficits in the exploratory behaviour of mice, as determined by a reduction in the number of entries and the time spent in the centre quadrant of the OF (inner white box on representative heat maps; Figure 1A). Notably, at day 21 there was a significant reduction in the number of entries in the centre of the OF (* *p* < 0.05; Figure 1B), which correlated with a reduction in the time spent in the centre at both day 14 (* *p* < 0.05) and day 21 (* *p* < 0.05) (Figure 1C). As expected, buspirone was able to prevent rotenone-induced deficits in exploratory behaviour (Figure 1B–C). 

Drug-induced effects on locomotor behaviour were also appraised by comparing the total distance travelled (Figure 1D) and the average speed (Figure 1E). Rotenone significantly impaired locomotion after only 14 days of treatment. There was a significant reduction in the total distance travelled at day 14 (* *p* < 0.05 vs. baseline) and day 21 (* *p* < 0.05) (Figure 1D), concurrent with a reduction in the average speed at both day 14 (* *p* < 0.05) and 21 (* *p* < 0.05) (Figure 1E). In rotenone-treated mice, 1 mg/kg of buspirone was unable to prevent deficits in locomotor behaviour. In fact, mice still displayed a significant reduction in the total distance travelled at both day 14 (* *p* < 0.05 vs. baseline) and day 21 (* *p* < 0.05 vs. baseline) (Figure 1D), which correlated with a slower average speed at day 21 (*** *p* < 0.001 vs. baseline; Figure 1E), compared to baseline measurements. However, higher concentrations of buspirone (3 and 10 mg/kg) prevented these locomotor deficits (Figure 1D,E). 

### 2.2. Buspirone Prevents the Loss of Dopaminergic Neurons in Rotenone-Treated Mice 

**Figure 2:**
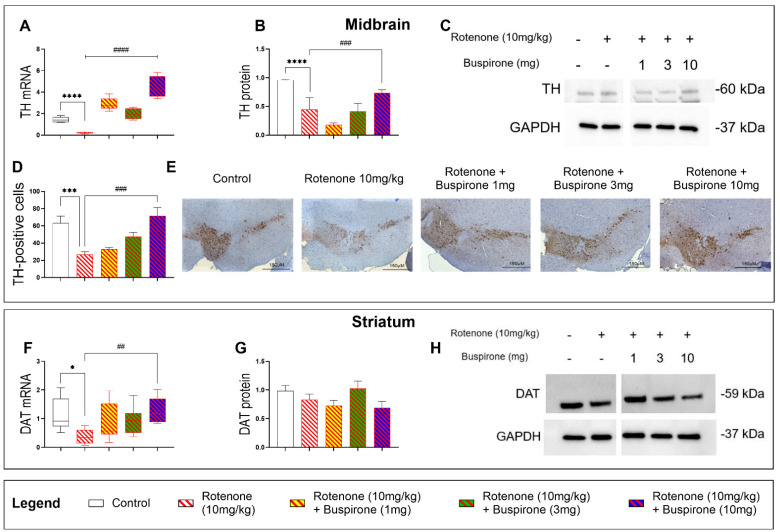
Buspirone at high dosages protects nigro-striatal dopaminergic neurons from degeneration. TH mRNA (**A**) and protein expression in the midbrain (**B**,**C**). Representative photomicrographs depicting TH immunoreactivity and semi-quantitative measurement of staining intensity in the *SNpc*, scale bar = 150 µM (**D**,**E**). Striatal DAT mRNA (**F**) and protein expression (**G**,**H**). Gene expression was measured by qRT-PCR and quantified using the ΔΔCt method after normalization to s18 (the housekeeping gene). qRT-PCR results are reported as mean fold changes with respect to vehicle-treated control mice. Protein expression was determined by Western blot and normalized to GAPDH (the loading control). Western blots are cropped and removed lanes depicting drug-treatment controls are included in Supplementary Figure S1. Densitometry results are presented as means ± S.E.M. TH-positive cells are reported as the percent of TH-positive cells normalized to the total number of nuclei ± S.E.M. Data represents 3–4 mice per group. * *p* < 0.05, *** *p* < 0.001, or **** *p* < 0.0001, ## *p* < 0.01, ### *p* < 0.001, or #### *p* < 0.0001, as determined by an ANOVA followed by Dunnett’s post-hoc test. TH, tyrosine hydroxylase; DAT, dopamine transporter; GAPDH, glyceraldehyde 3-phosphate dehydrogenase; kDa, Kilodalton; s18, ribosomal protein s18 gene.

To assess if buspirone protected dopaminergic neurons from the detrimental effects of rotenone, we analysed the expression two dopaminergic markers, tyrosine hydroxylase (TH) and dopamine transporter (DAT), in the midbrain and striatum, respectively (Figure 2). As expected, rotenone administration resulted in a significant reduction of TH mRNA (**** *p* < 0.0001 vs. vehicle; Figure 2A) and protein expression (**** *p* < 0.0001 vs. vehicle; Figure 2B,C) in the midbrain. Buspirone treatment upregulated the expression of TH transcripts in the midbrain, at all dosages, compared with rotenone-treated mice (**** *p* < 0.0001; Figure 2A). These results were associated with a dose-dependent increase in TH expression at the protein level, which was statistically significant at the highest dosage of buspirone (### *p* < 0.001 vs. rotenone-treated mice; Figure 2B). Immunohistochemical data corroborated these findings, as the significant reduction in the number of TH-positive cells in the SNpc (** *p* < 0.01 vs. vehicle) was prevented by buspirone in a dose-dependent manner, although results were significant only at the highest dosage tested (### *p* < 0.001, buspirone 10 mg/kg vs. rotenone-treated mice; Figure 2D,E). 

### 2.3. Buspirone Triggers Brain-Region-Specific Changes in the Expression of Glial Activation Markers 

**Figure 3:**
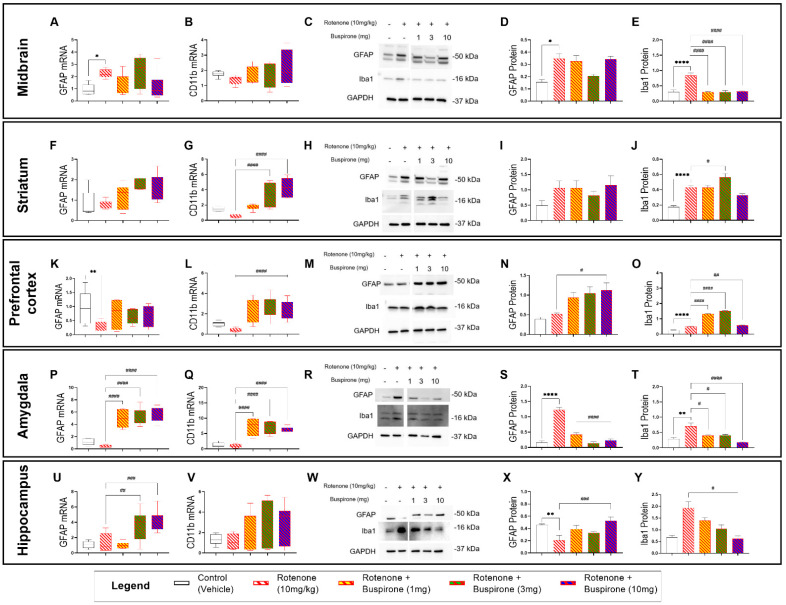
Buspirone triggers region-specific changes in the expression of glial activation markers. Real-time qPCR analyses of GFAP and CD11b in the midbrain (**A**,**B**), striatum (**F**,**G**), prefrontal cortex (**K**,**L**), amygdala (**P**,**Q**), and hippocampus (**U**,**V**) reported as mean fold changes calculated with the ΔΔCt method after normalization to s18 (the housekeeping gene). Western blot and densitometric analysis of GFAP and Iba1 in the midbrain (**C**–**E**), striatum (**H**–**J**), prefrontal cortex (**M**–**O**), amygdala (**R**–**T**), and hippocampus (**W**–**Y**). Western blots are cropped and removed lanes depicting drug-treatment controls are included in Supplementary Figure S3. Densitometric results are expressed as means ± S.E.M. All data represents 4–6 samples per treatment group. * *p* < 0.05, ** *p* < 0.01, or **** *p* < 0.0001, compared to vehicle-treated controls; or # *p* < 0.05, ## *p* < 0.01, ### *p* < 0.001, or #### *p* < 0.0001, compared to rotenone (10 mg/kg)-treated mice, as determined by an ANOVA followed by Sidak’s post-hoc test. GFAP, Glial Fibrillary Acidic Protein; Iba1, ionized calcium binding adapter molecule 1; s18, ribosomal protein s18 gene; GAPDH, Glyceraldehyde 3-phosphate Dehydrogenase; kDa, Kilodalton.

To determine if buspirone treatment prevented rotenone-induced activation of astrocytes and microglia, we measured the relative mRNA and protein expression of GFAP (astrocytic activation marker) and CD11b or Iba1 (microglial activation markers) in several brain regions (i.e., midbrain, striatum, prefrontal cortex, amygdala, and hippocampus). To our surprise, glial activation followed a region-specific pattern and was more pronounced in extra-nigral regions (Figure 3).

In the midbrain, GFAP mRNA and protein expression were increased in rotenone-treated mice (* *p* < 0.05 vs. vehicle; Figure 3A,C,D); however, co-treatment with buspirone failed to prevent GFAP gene and protein induction at all the dosages tested (*p* > 0.05 vs. rotenone). Striatal GFAP transcripts and proteins were not significantly affected by rotenone exposure or buspirone co-treatment, although expression levels were slightly increased when compared with controls (*p* > 0.05 vs. vehicle; Figure 3F,H,I). In the prefrontal cortex, GFAP transcripts were significantly downregulated in response to rotenone administration (** *p* < 0.01 vs. vehicle; Figure 3K), but not in animals that were co-treated with buspirone, where levels were comparable to controls (*p* > 0.05 vs. vehicle). In contrast, GFAP protein levels were not diminished by rotenone and were significantly increased in animals that received 10 mg/kg of buspirone (# *p* < 0.05; Figure 3M,N). In the amygdala, GFAP mRNA expression was slightly reduced by rotenone exposure (*p* > 0.05 vs. vehicle). Surprisingly, buspirone co-treatment caused a strong increase in GFAP transcripts at all dosages (#### *p* < 0.0001 vs. rotenone; Figure 3P). Conversely, GFAP protein expression was remarkably increased in the amygdala or rotenone-intoxicated mice (**** *p* < 0.0001 vs. vehicle) and significantly reduced by buspirone co-administration (#### *p* < 0.0001 vs. rotenone; Figure 3R,S). Hippocampal GFAP transcripts were not affected by rotenone (*p* > 0.05 vs. vehicle); however, mRNA levels were significantly increased when animals were co-administered with the two highest dosages of buspirone (## *p* < 0.01 and ### *p* < 0.0001 vs. rotenone, respectively; Figure 3U). GFAP protein expression in the hippocampus did not match mRNA data. In fact, GFAP protein levels were significantly reduced upon rotenone treatment (** *p* < 0.01 vs. vehicle) and increased in response to the highest dosage of buspirone (### *p* < 0.001 vs. rotenone; Figure 3W,X). 

Gene expression studies of the microglial marker CD11b across the different brain regions did not show any major effects of rotenone (*p* > 0.05 vs. vehicle; Figure 3B,G,L,Q,V), although some trends towards a reduction were seen in the striatum and prefrontal cortex. In contrast, buspirone co-treatment induced significant CD11b gene upregulation in the striatum (#### *p* < 0.0001 vs. rotenone at 3 and 10 mg/kg; Figure 3G), prefrontal cortex (#### *p* < 0.0001 vs. rotenone at 1, 3, and 10 mg/kg: Figure 3L), amygdala (#### *p* < 0.0001 vs. rotenone at 1, 3, and 10 mg/kg: Figure 3Q), and, to some extent, in the hippocampus, although not significantly (*p* > 0.05 vs. rotenone at 1, 3, and 10 mg/kg: Figure 3V). 

Protein expression of the microglial marker Iba1 showed a robust induction in the CNS following rotenone exposure, and levels were in great part prevented upon buspirone co-administration (Figure 3E,J,O,T,Y). In more detail, rotenone significantly increased Iba1 protein expression in the midbrain (**** *p* < 0.0001 vs. vehicle; Figure 3C,E), striatum (**** *p* < 0.0001 vs. vehicle; Figure 3H,J), prefrontal cortex (**** *p* < 0.0001 vs. vehicle, Figure 3M,O), amygdala (** *p* < 0.01 vs. vehicle; Figure 3R,T), and, to a minor extent, in the hippocampus, although not significantly (*p* > 0.05 vs. vehicle; Figure 3W,Y). 

In the midbrain, all dosages of buspirone prevented rotenone-induced Iba1 upregulations (#### *p* < 0.0001 vs. rotenone; Figure 3E). Within the striatum, Iba1 expression was not affected by either buspirone at 1 mg/kg or at 10 mg/kg; however, it was significantly increased at 3 mg/kg (# *p* < 0.05 vs. rotenone; Figure 3H,I). Buspirone also reduced Iba1 expression in the amygdala (# *p* < 0.05 at 1 and 3 mg/kg and #### *p* < 0.0001 at 10 mg/kg buspirone vs. rotenone; Figure 3R,T) and in the hippocampus, but only at the dosage of 10 mg/kg of buspirone (# *p* < 0.05 vs. rotenone; Figure 3Y). 

### 2.4. Buspirone Reduces Neuroinflammation and Promotes the Expression of Neurotrophic Factors 

**Figure 4:**
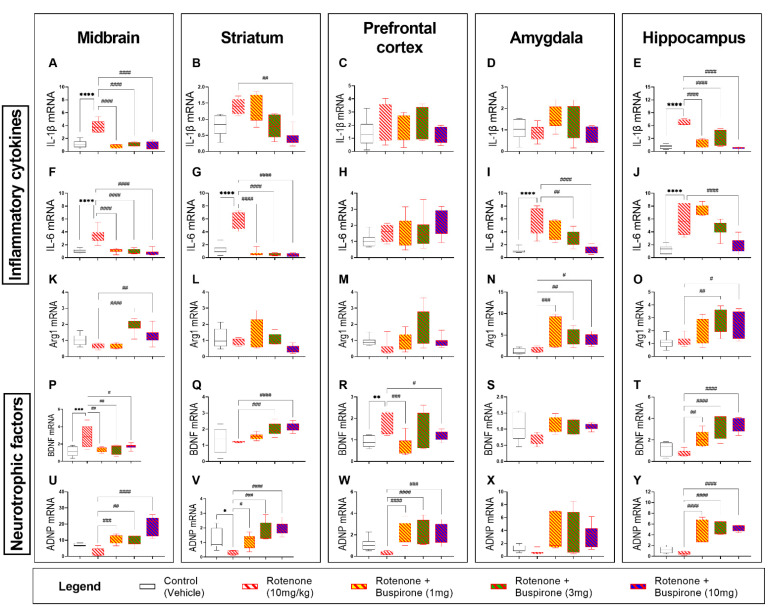
Buspirone reduces the expression of inflammatory cytokines and upregulates the expression of neurotrophic factors. Real-time qPCR analyses of the pro-inflammatory cytokines IL-1β (**A**–**E**) and IL-6 (**F**–**J**), the anti-inflammatory cytokine Arg1 (**K**–**O**), and the neurotrophic factors BDNF (**P**–**T**) and ADNP (**U**–**Y**) in the midbrain (box 1), striatum (box 2), prefrontal cortex (box 3), amygdala (box 4), and hippocampus (box 5) were reported as mean fold changes calculated with the ΔΔCt method after normalization to s18 (the housekeeping gene). All data represents 5–8 samples per treatment group. * *p* < 0.05, ** *p* < 0.01, *** *p* < 0.001, or **** *p* < 0.0001, compared to vehicle-treated controls; or # *p* < 0.05, ## *p* < 0.01, ### *p* < 0.001, or ####*p* < 0.0001, compared to rotenone (10 mg/kg)-treated mice, as determined by an ANOVA followed by Sidak’s post-hoc test. IL-1β, interleukin-1beta; IL-6, interleukin-6; Arg1, arginase 1; BDNF, brain-derived neurotrophic factor; ADNP, activity-dependent neuroprotective protein; s18, ribosomal protein s18 gene; GAPDH, Glyceraldehyde 3-phosphate Dehydrogenase; kDa, Kilodalton.

In view of the effects of buspirone in the glial compartment, we sought to interrogate the mRNA expression of several pro-inflammatory cytokines (IL-1β and IL-6) and the anti-inflammatory marker (Arg1), as well as that of two neurotrophic factors (BDNF and ADNP) after 21-days of systemic rotenone administration with or without buspirone co-administration by qRT-PCR (Figure 4). The systemic administration of rotenone (10 mg/kg) significantly induced the expression of IL-1β in the midbrain (**** *p* < 0.0001; Figure 4A) and hippocampus (**** *p* < 0.0001; Figure 4E), but not in the striatum, prefrontal cortex, or amygdala (*p* > 0.05 vs. vehicle; Figure 4B–D). Similarly, the expression of IL-6 was significantly upregulated in the midbrain (**** *p* < 0.0001; Figure 4F), striatum (**** *p* < 0.0001; Figure 4G), amygdala (**** *p* < 0.0001; Figure 4I), and hippocampus (**** *p* < 0.0001; Figure 4J), but not in the prefrontal cortex (*p* > 0.05; Figure 4C). 

Gene expression of the pro-inflammatory cytokines IL-1β and IL-6 were globally downregulated in response to buspirone co-treatment (Figure 4A–J). Both IL-1β (#### *p* < 0.0001; Figure 4A) and IL-6 (#### *p* < 0.0001; Figure 4F) were robustly downregulated in the midbrain compared with rotenone-treated mice. In the striatum, all buspirone dosages were able to reduce IL-6 expression (#### *p* < 0.0001; Figure 4G), but only the 10 mg/kg buspirone does was able to reduce IL-1β mRNA expression in the striatum (## *p* < 0.01 vs. rotenone-treated mice; Figure 4B). Similarly, in the hippocampus, 10 mg/kg of buspirone was required to fully prevent rotenone-driven upregulation of IL-6 expression (#### *p* < 0.0001; Figure 4J), whilst all buspirone dosages reliably attenuated IL-1β levels within the same brain region (#### *p* < 0.0001; Figure 4E). Both 3 and 10 mg/kg of buspirone significantly downregulated IL-6 expression in the amygdala (## *p* < 0.01 and #### *p* < 0.0001, respectively; Figure 4I). Buspirone treatment had no effects on IL-1β in the amygdala or on either pro-inflammatory cytokines in the prefrontal cortex (*p* > 0.05; Figure 4C,D,H). 

Real-time qPCR analyses revealed that rotenone administration did not alter the expression of the anti-inflammatory marker Arg1 in the brain (Figure 4K–O). Conversely, both 3 and 10 mg/kg of buspirone significantly increased Arg1 mRNA in the midbrain (#### *p* < 0.0001 and ## *p* < 0.01 vs. rotenone, respectively; Figure 4K) and hippocampus (## *p* < 0.01 and # *p* < 0.05, respectively; Figure 4O). In the amygdala, all buspirone treatment groups demonstrated an increase in Arg1 mRNA expression in response to drug co-treatment (### *p* < 0.001, ## *p* < 0.01 and # *p* < 0.05 at 1, 3, and 10 mg/kg, respectively; Figure 4N), whereas it had no ameliorative effects in the striatum and prefrontal cortex (*p* > 0.05 vs. rotenone; Figure 4L,M). 

The mRNA expression of the neurotrophic factors BDNF and ADNP was assessed in the CNS of mice exposed to rotenone and/or co-treated with buspirone. As shown, rotenone significantly increased BDNF transcripts in the midbrain and prefrontal cortex (*** *p* < 0.001 and ** *p* < 0.01 vs. vehicle, respectively; Figure 4P,R), but not in the striatum, amygdala, and hippocampus, where transcripts were marginally decreased (*p* > 0.05 vs. vehicle; Figure 4Q,S,T). The co-administration of buspirone to rotenone-treated mice partly reversed the effects of the toxicant on gene expression. In the midbrain, it significantly reversed BDNF gene induction at all the dosages tested (## *p* < 0.01 at 1 and 3 mg/kg and # *p* < 0.05 at 10 mg/kg; Figure 4P). Striatal BDNF mRNA were significantly increased when buspirone was used at 3 and 10 mg/kg (### *p* < 0.001 and #### *p* < 0.0001 vs. rotenone; Figure 4Q), an effect that was seen at all dosages in the hippocampus (## *p* < 0.01 at 1 mg/kg and #### *p* < 0.0001 vs. rotenone at 3 and 10 mg/kg; Figure 4T). In the prefrontal cortex, drug co-treatment reliably reduced BDNF gene expression (### *p* < 0.001 at 1 mg/kg and # *p* < 0.05 at 10 mg/kg, respectively; Figure 4R). In the amygdala, BDNF gene expression was not affected by rotenone or buspirone co-administration (*p* > 0.05; Figure 4S). 

A global trend towards a reduction of ADNP transcripts was observed in the CNS of animals treated with rotenone, although this was significant only in the striatum (* *p* < 0.05 vs. vehicle; Figure 4U–Y). In contrast, buspirone robustly increased ADNP mRNA in the CNS regions tested. In the midbrain, buspirone increased ADNP (### *p* < 0.001, ## *p* < 0.01 and #### *p* < 0.0001 vs. rotenone at 1, 3 and 10 mg/kg; Figure 4U). Similarly, in the striatum, both 1, 3, and 10 mg/kg of buspirone significantly increased the expression of ADNP (# *p* < 0.05, ## *p* < 0.01, and #### *p* < 0.0001, respectively; Figure 4V). Additionally, all buspirone treatments promoted the upregulation of ADNP in the prefrontal cortex (#### *p* < 0.0001, #### *p* < 0.0001, and ### *p* < 0.001 at 1, 3, and 10 mg/kg buspirone, respectively; Figure 4W) and hippocampus (#### *p* < 0.0001; 1, 3, and 10 mg/kg buspirone, respectively; Figure 4Y), whereas only a trend was seen in the amygdala (*p* > 0.05 vs. rotenone; Figure 4X). 

### 2.5. Buspirone Dampens Rotenone-Induced Oxidative Stress in Distinct Brain Regions

**Figure 5:**
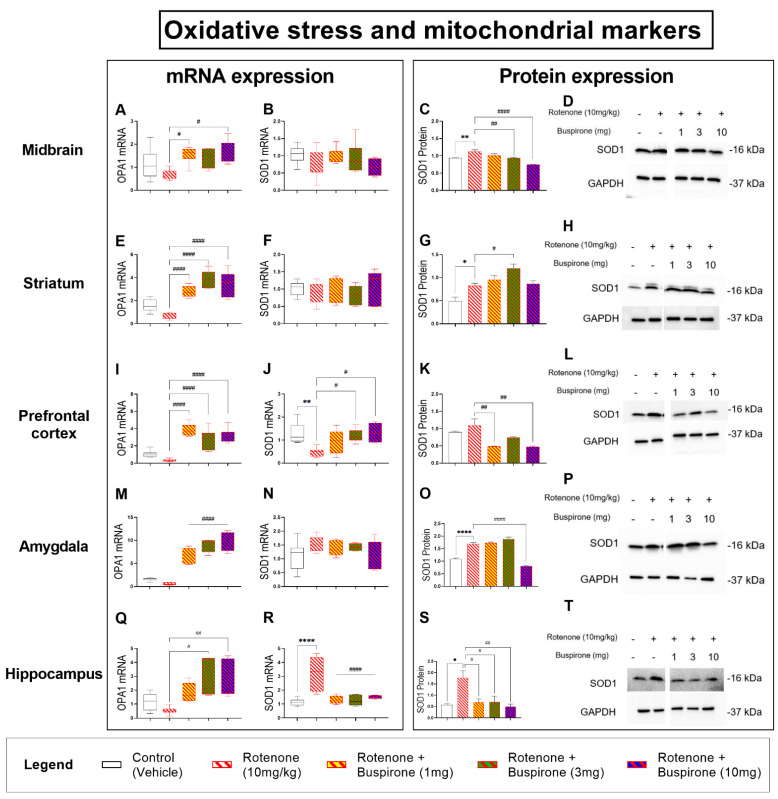
Buspirone dampens rotenone-induced oxidative stress in the brain. The mitochondrial marker, OPA1, was analysed to determine rotenone-induced mitochondrial damage and the antioxidant enzyme SOD1 was investigated as an indirect measure of oxidative stress. Real-time qPCR analyses of OPA1 and SOD1 mRNA expression in the midbrain (**A**,**B**), striatum (**E**,**F**), prefrontal cortex (**I**,**J**), amygdala (**M**,**N**), and hippocampus (**Q**,**R**) following the administration of rotenone (10 mg/kg) and/or buspirone (1, 3, or 10 mg) daily for 21 days. Fold-changes were calculated using the ΔΔCt method after normalization to s18 (ribosomal protein s18 gene), the housekeeping gene. Each data point represents the mean value from n = 5–8 mice per each group. Representative Western blots and densitometry of SOD1 protein expression in the midbrain (**C**,**D**), striatum (**G**,**H**), prefrontal cortex (**K**,**L**), amygdala (**O**,**P**), and hippocampus (**S**,**T**). Protein expression was normalized to GAPDH, the loading control. Western blots are cropped and removed lanes depicting drug-treatment controls are included in Supplementary Figure S5. Densitometric results are expressed as means ± S.E.M from n = 5–8 mice per each group. * *p* < 0.05, ** *p* < 0.01 or **** *p* < 0.0001, compared to vehicle-treated controls; or # *p* < 0.05, ## *p* < 0.01 or ####*p* < 0.0001, compared to rotenone (10 mg/kg)-treated mice, as determined by an ANOVA followed by Sidak’s post-hoc test. OPA1, Mitochondrial dynamin like GTPase; SOD1, superoxide dismutase 1; s18, ribosomal protein s18 gene; GAPDH, glyceraldehyde 3-phosphate dehydrogenase; kDa, Kilodalton.

Rotenone is a known mitochondrial complex I inhibitor that is able to increase the levels of reactive oxygen species, promoting oxidative stress and mitochondrial damage. To determine if buspirone could prevent mitochondrial damage, we assessed mitochondrial function by analyzing OPA1 mRNA expression and the expression of the antioxidant enzyme SOD1, as an indirect measure of oxidative stress, throughout the brain (Figure 5). 

Analyses of OPA1 transcripts revealed that rotenone attenuated gene expression throughout the CNS, but not significantly (*p* > 0.05; Figure 5A,E,I,M,Q). Buspirone treatments promoted the expression of OPA1 mRNA, specifically in the midbrain (# *p* < 0.05 vs. rotenone at 1 and 10 mg/kg, Figure 5A), striatum (#### *p* < 0.0001; Figure 5E), prefrontal cortex (#### *p* < 0.0001; Figure 5I), amygdala (#### *p* < 0.0001; Figure 5M), and hippocampus (# *p* < 0.05 and ## *p* < 0.01 at 3 and 10 mg/kg; Figure 5Q). 

Real-time qPCR analysis of SOD1 mRNA levels did not show any changes in the midbrain and striatum after treatment with rotenone and/or co-treatment with buspirone (Figure 5B,F). However, Western blots to measure SOD1 protein levels in these same brain regions showed significant increases following rotenone exposure (** *p* < 0.01 and * *p* < 0.05 vs. vehicle, respectively), which were prevented using buspirone (midbrain: ## *p* < 0.01 and #### *p* < 0.0001 vs. rotenone at 3 and 10 mg/kg; striatum: # *p* < 0.05 vs. rotenone; Figure 5C,D,G,H). In the prefrontal cortex, SOD1 mRNA expression was significantly reduced by rotenone (** *p* < 0.01 vs. vehicle) and rescued by 3 and 10 mg/kg buspirone (# *p* < 0.05 for both; Figure 5J). However, results in this brain region were not corroborated by protein expression analyses. Prefrontal SOD1 protein expression was slightly increased by rotenone (*p* > 0.05) and diminished following buspirone treatment (## *p* < 0.01 at both 1 and 10 mg/kg; Figure 5K,L). Similar to the midbrain and striatum, SOD1 mRNA were not affected by rotenone and/or buspirone treatments in the amygdala (Figure 5N). However, at the protein level, we observed a significant increase of SOD1 expression after rotenone exposure (**** *p* < 0.0001 vs. vehicle), which was prevented by co-treatment with the highest dosage of buspirone (#### *p* < 0.0001 vs. rotenone; Figure 5O,P). Lastly, hippocampal SOD1 mRNA levels were significantly increased by rotenone (**** *p* < 0.0001 vs. vehicle) and fully prevented by co-administration with buspirone at all dosages (#### *p* < 0.0001 vs. rotenone; Figure 5R). Transcript results were corroborated by protein findings, as SOD1 protein expression was induced by rotenone (* *p* < 0.05 vs. vehicle) and mitigated by buspirone (# *p* < 0.05 vs. rotenone at 1 and 3 mg/kg, ## *p* < 0.01 vs. rotenone at 10 mg/kg; Figure 5S,T). 

### 2.6. Buspirone Prevents Dysregulations of the PACAP and VIP Neuropeptides in the Brains of Rotenone-Treated Mice 

**Figure 6:**
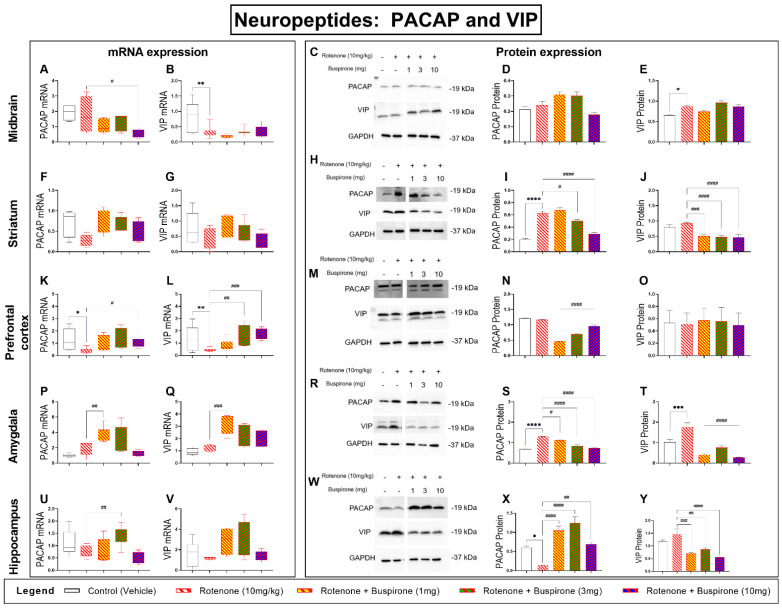
Buspirone prevents PACAP and VIP neuropeptide dysregulations in the brain of rotenone-treated mice. Real-time qPCR analyses of PACAP and VIP mRNA expression in the midbrain (**A**,**B**), striatum (**F**,**G**), prefrontal cortex (**K**,**L**), amygdala (**P**,**Q**), and hippocampus (**U**,**V**) following the administration of rotenone (10 mg/kg) and/or buspirone (1, 3, or 10 mg) daily for 21 days. Fold-changes were calculated using the ΔΔCt method after normalization to s18 (ribosomal protein s18 gene), the housekeeping gene. Each data point represents the mean value from *n* = 5–8 mice per each group. Representative Western blots and densitometry of PACAP and VIP protein expression in the midbrain (**C**–**E**), striatum (**H**–**J**), prefrontal cortex (**M**–**O**), amygdala (**R**–**T**), and hippocampus (**W**–**Y**). Protein expression was normalized to GAPDH, the loading control. Western blots are cropped and removed lanes depicting drug-treatment controls are included in Supplementary Figure S6. Densitometric results are expressed as means ± S.E.M from n = 5–8 mice per each group. * *p* < 0.05, ** *p* < 0.01, *** *p* < 0.001, or **** *p* < 0.0001, compared to vehicle-treated controls; or # *p* < 0.05, ## *p* < 0.01, ### *p* < 0.001, or #### *p* < 0.0001, compared to rotenone (10 mg/kg)-treated mice, as determined by an ANOVA followed by Sidak’s post-hoc test. PACAP, pituitary adenylate cyclase-activating peptide; VIP, vasoactive intestinal peptide; s18, ribosomal protein s18 gene; GAPDH, glyceraldehyde 3-phosphate dehydrogenase; kDa, Kilodalton.

The neuropeptides PACAP and VIP are involved in a range of neuroprotective and immune modulatory functions in the CNS [21]. We have previously shown that the genetic blockade of the D3R promotes the expression of PACAP [22]. To determine if buspirone, a D3R antagonist, altered the expression of these peptides during rotenone intoxication, we analysed their gene and protein expression throughout the brain (Figure 6). 

In the midbrain, rotenone exposure alone had no effects on PACAP mRNA (*p* > 0.05 vs. vehicle); however, the highest dosage of buspirone reduced gene expression (# *p* < 0.05 vs. rotenone; Figure 6A). Protein analyses did not show significant changes in PACAP protein expression in response to rotenone and/or buspirone co-treatment (*p* > 0.05; Figure 6C,D). In the striatum, PACAP mRNA were slightly reduced, and co-treatment with buspirone prevented these effects, although not significantly (*p* > 0.05 vs. rotenone; Figure 6F). In contrast, PACAP protein expression was robustly increased by rotenone in the striatum (**** *p* < 0.0001 vs. vehicle) and buspirone reduced the expression at 3 and 10 mg/kg (# *p* < 0.05 and #### *p* < 0.0001 vs. rotenone; Figure 6H,I). In the prefrontal cortex, PACAP transcripts were downregulated in response to rotenone (* *p* < 0.05 vs. vehicle). Co-treatment with buspirone significantly increased gene expression (# *p* < 0.05 vs. rotenone; Figure 6K). At the protein level, PACAP showed no changes after rotenone exposure; however, buspirone co-treatment significantly reduced PACAP protein expression (#### *p* < 0.0001 vs. rotenone; Figure 6M,N). In the amygdala, rotenone did not modify the mRNA expression of PACAP (*p* > 0.05 vs. vehicle); however, a significant induction was seen after 1 mg/kg buspirone co-administration (## *p* < 0.01 vs. rotenone; Figure 6P). In contrast, PACAP protein analyses showed that rotenone strongly increased protein expression (**** p < 0.0001 vs. vehicle) and levels were remarkably diminished in the amygdala of mice co-treated with both rotenone and buspirone (# *p* < 0.05 at 1 mg/kg and #### *p* < 0.0001 vs. rotenone at 3 and 10 mg/kg, respectively; Figure 6R,S). Hippocampal PACAP transcripts were only modestly reduced by rotenone (*p* > 0.05 vs. vehicle) and significantly increased in response to 3 mg/kg of buspirone (## *p* < 0.01 vs. rotenone; Figure 6U). At the protein level, PACAP was significantly reduced by rotenone (* *p* < 0.05 vs. vehicle) and reliably increased by buspirone (#### *p* < 0.0001 at 1 and 3 mg/kg, ## *p* < 0.01 vs. rotenone at 10 mg/kg; Figure 6W,X). 

Analyses of VIP transcripts in the midbrain revealed a significant reduction in gene expression after rotenone exposure (** *p* < 0.01 vs. vehicle), which was not ameliorated by buspirone co-treatment (*p* > 0.05 vs. rotenone; Figure 6B). However, Western blots did not support these results, as VIP expression was upregulated by rotenone (* *p* < 0.05 vs. vehicle) and not further affected by buspirone (*p* > 0.05 vs. rotenone; Figure 6C,E). We did not observe any significant transcript changes in the striatum (*p* > 0.05; Figure 6G), whereas protein results showed that buspirone significantly reduced VIP expression (### *p* < 0.001 at 1 mg/kg, #### *p* < 0.0001 vs. rotenone at 3 and 10 mg/kg; Figure 6H,J). In the prefrontal cortex, VIP mRNA was reduced by rotenone (** *p* < 0.01 vs. vehicle) and returned to control levels upon buspirone co-treatment (## *p* < 0.01 and ### *p* < 0.001 at 3 and 10 mg/kg, respectively; Figure 6L). However, these results were not confirmed by Western blots, as VIP expression was unchanged in response to treatments (*p* > 0.05; Figure 6M,O). In the amygdala, VIP mRNA expression was not affected by rotenone treatment (*p* > 0.05 vs. vehicle); however, transcripts were upregulated by buspirone (### *p* < 0.001 vs. rotenone; Figure 6Q). Instead, VIP protein expression was upregulated by rotenone (*** *p* < 0.001 vs. vehicle) and robustly reduced upon buspirone co-administration (#### *p* < 0.0001 vs. rotenone at all dosages; Figure 6R,T). Finally, hippocampal VIP mRNA levels were unchanged after rotenone and/or buspirone co-treatment (*p* > 0.05; Figure 6V). However, VIP protein expression was reduced in the hippocampus of mice co-treated with rotenone and buspirone (### *p* < 0.001 at 1 mg/kg, ## *p* < 0.01 at 3 mg/kg and #### *p* < 0.0001 vs. rotenone at 10 mg/kg; Figure 6W,Y). 

## 3. Discussion

Pharmacological blockade of the D3R has previously been shown to reduce inflammation and, consequently, slow disease progression in animal models of PD [11]. Interestingly, computational analyses have identified buspirone as a drug endowed with strong off-target function as D3R antagonist [16]. In addition, we previously demonstrated that buspirone treatment reliably attenuated LPS-triggered microglial polarization in BV2 cells, an effect that was not seen in D3R knockout (D3R^−/−^) microglia [17]. This led us to test the efficacy of buspirone to reduce inflammation and promote neuroprotection in a rotenone mouse model of PD. 

To our knowledge, this is the first study to investigate the ability of buspirone to prevent inflammation and promote neuroprotection in a model of PD. We report that buspirone prevented rotenone-induced deficits in locomotor and exploratory behaviour, and dose-dependently rescued dopaminergic degeneration in the *substantia nigra pars compacta,* midbrain, and striatum. Interestingly, buspirone also prevented glial activation in a brain-region-specific pattern. Concurrently, the drug robustly prevented the induction of pro-inflammatory cytokines by rotenone and increased the expression of neurotrophic and anti-inflammatory factors, suggesting that buspirone is able to afford some of its beneficial effects by modulating the immune response and by promoting the release of protective factors. Additionally, buspirone restored the levels of the mitochondrial marker OPA1 and the antioxidant enzyme SOD1, as well as prevented the disruptions to the two neuroprotective and immune modulatory peptides PACAP and VIP throughout the brain. 

Several studies have described behavioural deficits in mice after systemic administration or rotenone [23,24,25,26]. These studies, consistent with current findings using 10 mg/kg of rotenone, indicate that a three-week induction regime using this dosage of pesticide is sufficient to attain locomotor impairments consistent with PD-like pathology. Previous studies on D3R^−/−^ mice revealed that this dopamine receptor subtype mediates inhibitory effects on aversive learning [27] and both spontaneous and forced ethanol consumption in mice [16]. In this study, we did not observe any specific changes in general locomotor and exploratory behavior, nor in the expression of dopaminergic markers in mice treated with buspirone alone (Supplementary Figures S1 and S2), in contrast to previous reports from other laboratories using rats [28]. However, when the drug was co-administered with rotenone at the highest dosages (3 and 10 mg/kg), it successfully prevented the behavioural impairments caused by the insecticide. As such, it is conceivable that any preventative effect of buspirone on rotenone-induced behavioural impairments was likely to be attributed to its widespread restorative capacity in the CNS. Interestingly, similar anxiolytic and motor effects have been previously reported in mice treated with minocycline, a tetracycline antibiotic endowed with anti-inflammatory functions, further suggesting that agents able to attenuate inflammation may also be effective in preventing any associated behavioural deficits [20]. These effects are likely to be attributed to buspirone-mediated D3R antagonistic activity, as our results corroborate prior evidence of reduced anxiety-like behaviour in D3R^−/−^ mice [29]. Unfortunately, buspirone’s ameliorative effects on locomotor activity in rodents haven’t been successfully translated into a clinical setting, as PD patients subjected to a buspirone drug treatment protocol showed poor outcomes in terms of anxiolytic response [30]. This species-specific diversity in drug response could be due to the different D3R vs. D2R distribution in the CNS between mice and humans. Additionally, given that buspirone also binds to D2R (with much lower affinity than D3R) to antagonize receptor activation [30], it is also possible that a drug-induced D2R blockade in humans (but not in rodents) may elicit some anti-psychotic effects that, however, are not accompanied by improvements on motor function, as shown in other studies [31]. It is the high homology between D2R and D3R that renders the development of specific D3R antagonists challenging and often results in mixed effects. Further refinement of the drug to enhance receptor selectivity may be a viable strategy to overcome this unwanted effect. However, it should be noted that the lack of motor improvements in PD patients is not necessarily paralleled by the lack of other critical disease-modifying effects of the drug, such as the reduced neuroinflammation and increased trophic support reported in this study. In addition, we cannot exclude that some of buspirone’s inherent activities may also promote some degree of glial activation and stimulate the expression of trophic factors in certain CNS regions (Supplementary Figures S3–S6), whose significance warrants additional investigations. Furthermore, the neuroprotective effects can also be partly attributed to its canonical 5HT1a agonist activity, which may act synergistically to the D3R to improve the neurochemical disbalances seen in PD. 

The landmark study by Elgueta and colleagues [11] demonstrated that pharmacological antagonism of D3R reduced the extent of dopaminergic degeneration in an MPTP model of PD [11]. Here, we report a similar dose-dependent protection of dopaminergic neurons in response to 10 mg/kg of buspirone. This finding corroborates the hypothesis that high dosages of buspirone are required to achieve enough D3R occupancy [32]. The protection of dopaminergic neurons is suggested to be secondary to buspirone’s ability to reduce neuroinflammation. Accordingly, we analysed the expression of glial activation markers, inflammatory cytokines, and neurotrophic factors in distinct brain regions to determine how buspirone modulated the immune response in the brain. 

Gao and colleagues [18] revealed that the presence of microglia greatly enhanced the neurodegenerative and neurotoxic effects of rotenone by increasing the susceptibility of neuron-enriched cultures to the toxin. In line with this study, we show that rotenone treatment induced the upregulation of microglial activation markers and pro-inflammatory cytokines. Previously, our laboratory has shown that D3R gene deletion and/or buspirone treatment prevented LPS-triggered microglial activation [17]. Here, we sought to determine if buspirone could exert similar effects in vivo. Therefore, we interrogated the expression of glial activation markers along with that of pro-inflammatory cytokines and neurotrophic factors. In contrast to our in vitro study, in vivo we observed a drug-induced reduction of Iba1 expression in the midbrain and amygdala, and an increase in the prefrontal cortex compared with mice receiving rotenone alone. Unexpectedly, these results were accompanied by a global increase in CD11b transcripts, which contrasts with the Iba1 response to the anxiolytic drug. These differences in the expression of glial activation markers could be due to the acquisition of differing microglial phenotypes during the course of PD. It is known that activated microglia exist in two main phenotypes, M1, considered to be pro-inflammatory, and M2, considered to be anti-inflammatory [33]. Depending on disease severity and the local CNS inflammatory state, microglia may present as heterogeneous subpopulations that may or may not benefit from the anti-inflammatory effects of the drug, although this remains to be ascertained. This theory would explain why we saw a region-specific variability in the expression of glial activation markers. 

Buspirone also produced a robust downregulation of the pro-inflammatory cytokines IL-1β and IL-6. This was accompanied by a global upregulation of the anti-inflammatory marker Arg1, as well as a remarkable increase in the expression of neuroprotective and neurotrophic/growth factors, all known to exert a range of protective effects in the CNS [34]. Rotenone is known to reduce the expression of BDNF in vulnerable CNS regions [35]. This was partly seen in the hippocampus and amygdala, whereas levels were increased in the midbrain and prefrontal cortex. Irrespective of how BDNF transcripts were regulated by rotenone in the different CNS sites, buspirone prevented these changes. These results suggest that the drug is able to promote neuroplasticity, cell survival, and perhaps axonal growth during rotenone toxicity [36]. This is corroborated by the significant and robust induction of ADNP in all the brain regions we studied. ADNP is also essential in neuronal survival and brain formation, and is dysregulated in neurodegenerative and neuroinflammatory diseases [37]. The combined cytokine and neurotrophic profiles reported in this study indicate that buspirone favours the adoption of the M2 phenotype that promotes the dampening of neuroinflammation, CNS repair, and neuroprotection [38]. This is in agreement with our in vitro study that showed that D3R^−/−^ and/or buspirone treatment inhibited M1 markers, including NO, NOS2, IL-1β, and TNF-α [17]. However, microglia are not the only glial cell type mediating the immune response. They work closely with astrocytes; however, more studies are needed to elucidate the role of buspirone on astrocytes. We report minimal changes in the expression of the astrocytic marker, GFAP. This correlates with analysis of GFAP-positive cells and morphology in D3R^−/−^ mice that did not differ to wild-type animals [39], suggesting that the anti-inflammatory effect observed is primarily the result of buspirone’s activity on microglia and, perhaps, on other immune cells. However, it was shown that the neuroprotective effects of 5HT1a agonism are mediated by astrocytes, which prevent the dopaminergic degeneration in parkinsonian mice [40]. This suggests that the 5HT1a agonist activity of buspirone may also contribute to the neuroprotective effects reported in this study. 5HT1a agonists, including buspirone, have been reported as effective neuroprotective agents in traumatic brain injury [41]. The role of the serotonin system in PD is being revealed, with some studies suggesting the 5HT1a receptor could be a target alone for improving both motor function [42] and microglial responses [43]. 

Rotenone toxicity is mediated by its inhibitory action on mitochondria, which ultimately results in oxidative stress [44]. To determine if the effects of buspirone were due to rotenone intoxication, we analysed the expression of OPA1, a mitochondrial marker, and SOD1, an antioxidant enzyme, as indirect evidence of oxidative stress. As expected, rotenone significantly reduced the expression of OPA1, indicating mitochondrial dysfunction. This was also verified by the upregulation of SOD1 throughout the various CNS regions. The widespread distribution of rotenone intoxication suggests this model may be capturing additional prodromal dysfunctions of the disease, as changes in extra-nigral regions correlated with changes in CNS regions that are associated with some of the non-motor symptoms that usually remain silent or do not clinically manifest until motor symptoms appear [45,46,47]. This could also explain the lack of glial activation, which is commonly observed in the late stages of the disease due to chronic neuroinflammation. 

PACAP and VIP have both been shown to promote neuroprotection and reduce inflammation in several models of PD [48]. Notably, in primary rat mesencephalic neuron-glia cultures, PACAP prevented microglial polarization and protected against LPS-induced DAergic neurotoxicity [49]. This aligns with our recent study demonstrating the ability of these peptides to reduce LPS-induced inflammation and promote unique microglial phenotypes in vitro [50]. We have previously shown that D3R deletion enhances the expression of theses peptides [22]. The upregulation of neuroprotective growth factors and peptides by buspirone provides additional evidence of its neuroprotective activity and therapeutic potential in neurodegenerative diseases; however, further investigations on the exact role exerted by these protective agents with respect to the specific brain regions remains a topic of further investigation. 

## 4. Materials and Methods

### 4.1. Animal Experiments 

Seventy-two 7-week-old C57BL/6 male mice were purchased from ARC (Perth, WA, Australia) (Table 1). Mice were housed in individually ventilated cages (4 mice per cage), under normal 12:12 h light/dark cycle, with access to food and water ad libitum. All experiments were conducted in line with the Australian Code of Practice for the Care and Use of Animals for Scientific Purposes. All animal experiments were approved by the University of Technology Sydney Animal Care and Ethics Committee (ETH19-3322, approved on the 10 May 2019). 

### 4.2. Experimental Protocol

**Figure 7:**
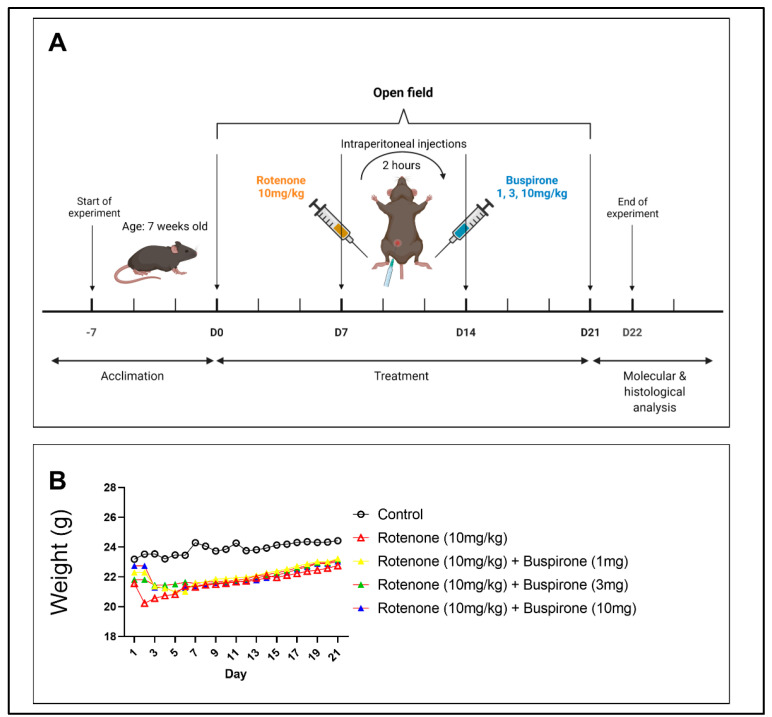
Experimental timeline. (**A**) Schematic of experimental timeline of injection regimen. Seven-week-old C57BL/6 male mice were allowed to acclimate for one week. Mice were exposed to daily intraperitoneal injections of rotenone (10 mg/kg), followed 2 h later by 1, 3, or 10 mg of buspirone for 21 days. At baseline, mice were subjected to the open field test for locomotor and exploratory behavioural assessment, and again on days 7, 14, and 21. On day 22, mice were euthanised and brains were collected for molecular and histological analysis. (**B**) Mice were weighed daily to monitor health and well-being. Data are represented as a daily average. N = 8–12 mice per group.

Rotenone was prepared as a stock solution in 0.1% DMSO diluted in 0.1% saline. The 0.1% DMSO was added to Parkinson’s disease saline treatment groups (Groups 1, 3–5) as a control. Buspirone was prepared as a stock solution in 0.1% saline. The experimental regime (Figure 7) lasted for 21 days, and involved daily intraperitoneal injections (i.p) of rotenone or saline, followed 2 h later by buspirone or saline, also administered by intraperitoneal injection. Mice were weighed daily and were subjected to the open field test every 7 days to assess locomotor and exploratory behavior. On day 22, mice were sacrificed and brains were collected. Brains were either snap frozen for molecular analysis or fixed in 4% paraformaldehyde for immunohistochemical analysis. Brains for molecular analysis were micro dissected into the following regions: prefrontal cortex, striatum, amygdala, hippocampus, and midbrain. 

### 4.3. Open Field

The open field (OF) test was performed every 7 days. Baseline measurements were performed prior to any intervention. Animals were acclimated in the testing room for 30 min for habituation. The OF test was conducted in the dark using a square box made of grey Plexiglass plastic (30 × 30 × 30 cm). Individually, mice were placed in the centre of the OF and allowed to freely explore for 5 min while being recorded. The OF was cleaned thoroughly between each mouse to eliminate excretions and any odour cues using 70% ethanol and allowed to air dry. The FIJI/ImageJ Plugin Mouse Behavioural Analysis Toolbox (MouBeAT) software ver. 1.00 [51] was used to analyse videos. Locomotor activity was determined by the total distance travelled and average speed of the mice, as calculated by MouBeAt. Exploratory behavior was defined as the number of times a mouse entered the centre of the OF and the time spent in the centre. 

### 4.4. Real-Time Quantitative Polymerase Chain Reaction (RT-qPCR)

Total RNA was extracted using TRI-reagent and precipitated with 2-propanol following established protocols [52]. Single-stranded cDNA was synthesized using the Tetro cDNA synthesis kit (Bioline, Sydney, NSW, Australia) as per the manufacturer’s instructions. Real-time qPCR was performed to analyse the mRNA levels of 12 genes (Table 2). The ribosomal protein 18S was used as the housekeeping gene. Each reaction consisted of 3 μL of cDNA (final concentration 100 ng), 5 μL iTaq Universal SYBR Green Master Mix (BioRad, VIC, Australia), and 0.8 μL of forward and reverse primers. To examine changes in expression, the mean fold changes of each sample was calculated using the ΔΔCt method as previously described [17]. PCR product specificity was evaluated by melting curve analysis, with each gene showing a single peak (data not shown). 

### 4.5. Western Blot 

Protein was extracted by homogenizing tissues in radioimmunoprecipitation assay (RIPA) buffer as previously described, and quantified using the bicinchoninic acid assay (Pierce BCA Protein Assay Kit, ThermoFisher Scientific, VIC, Australia). Next, 30 μg of protein was separated by SDS-polyacrylamide gel electrophoresis (SDS-PAGE) using 4–20% Mini-PROTEAN TGX Stain-Free Gels (15 well, BioRad, VIC, Australia). The Precision Plus Protein Prestained Standard in All Blue (BioRad, VIC, Australia) was included for comparison. Transfer to a PVDF membrane was performed using the semi-dry method (BioRad Trans-Blot Turbo Transfer System). Primary antibodies were incubated overnight in 5% skim milk in TBST blocking solution at 4 °C. Antibodies and dilutions are summarized in Table 3. Membranes were incubated in secondary antibody 1 hr at RT. Western blots were visualized using chemiluminescence BioRad Clarity Western ECL Blotting Substrate Solution. Images were acquired using the BioRad ChemiDoc MP System. Images were analysed using Fiji ImageJ and ratios normalized to GAPDH which was used as a loading control. 

### 4.6. Immunohistochemistry

Brains were fixed in 4% paraformaldehyde at 4 °C for 48 h before dehydration and embedding in paraffin. Next, 5 μM thick coronal sections were cut with a microtome and mounted on glass slides. The sections were deparaffinised in xylene and rehydrated through decreasing concentrations of ethanol. Dopaminergic neurons were labeled with TH. The immunoreactivity of TH was visualized using the Rabbit-specific HRP/DAB (ABC) Detection IHC Kit (ab64261, Abcam, VIC, Australia) according to the manufacturer’s protocol. Negative controls were created for all regions analysed by not incubating with the primary antibody. Hematoxylin was used as a counterstain to visualize nuclei. Sections were dehydrated in increasing concentrations of ethanol and xylene before being mounted. Images were taken on a ZEISS AxioScan.Z1 (Carl Zeiss Australasia, NSW, Australia) at ×20 magnification. Fiji ImageJ was used for the semi-quantification of DAB-positive cells normalized to nuclei (hematoxylin stain) [53]. 

### 4.7. Statistical Analysis 

All data are reported as the mean ± SEM. Statistical analyses were calculated using GraphPad Prism software v. 9.0.2. (GraphPad Software, La Jolla, CA, USA). Comparisons between two or more groups were analysed by ANOVA followed by Dunnett’s or Sidak’s post-hoc tests, as appropriate. *p*-values ≤ 0.05 were considered statistically significant. 

## 5. Conclusions

In conclusion, our results corroborate the idea that D3R receptor blockage may serve as a neuroprotective and anti-inflammatory mechanism to attenuate PD burden. Buspirone effectively prevented rotenone-induced behavioural deficits and dose-dependently protected dopaminergic neurons from the detrimental effects of rotenone intoxication. Buspirone mitigated rotenone toxicity throughout the brain, mainly by alleviating mitochondrial dysfunction, reducing inflammation, and promoting the expression of neuroprotective factors, including anti-inflammatory cytokines, neurotrophic and growth factors, and neuropeptides. Overall, this evidence suggests that buspirone may be a viable candidate for drug repurposing, as its anti-inflammatory and neuroprotective effects may prevent disease severity and progression in patients with neurodegenerative/neuroinflammatory conditions such as PD. 

## Figures and Tables

**Table 1:** Summary of treatment groups included in this study.

Group	Parkinson’s Disease	Buspirone
1	Saline (vehicle)	Saline (vehicle)
2	Rotenone 10 mg/kg	Saline (vehicle)
3 ^1^	Saline (vehicle)	Buspirone 1 mg/kg
4^1^	Saline (vehicle)	Buspirone 3 mg/kg
5 ^1^	Saline (vehicle)	Buspirone 10 mg/kg
6	Rotenone 10 mg/kg	Buspirone 1 mg/kg
7	Rotenone 10 mg/kg	Buspirone 3 mg/kg
8	Rotenone 10 mg/kg	Buspirone 10 mg/kg

^1^ Buspirone control groups (groups 3–5) are included as Supplementary Figures S1–S6.

**Table 2:** List of primer sets used in real-time qPCR analysis. Forward and reverse primers were selected from the 5′ and 3′ region of each gene. The expected length of each amplicon is indicated in the right column.

Accession #	Gene	Primer Sequence (5′-3′)	Length (bp)
NM_009377.3	Tyrosine hydroxylase (TH)	Fwd GCCCTACCAAGATCAAACCTAC Rev ATACGAGAGGCATAGTTCCTGA	93
NM_010020.3	Dopamine transporter (DAT)	Fwd ATGACATCAAGCAGATGACTGG Rev CACGACCACATACAGAAGGAAG	95
NM_001131020.1	Glial fibrillary acidic protein (GFAP)	Fwd GAGATTCGCACTCAATACGAGG Rev CTGCAAACTTAGACCGATACCA	79
NM_001082960.1	CD11b	Fwd GAGCAGGGGTCATTCGCTAC Rev GCTGGCTTAGATGCGATGGT	94
NM_008361.4	Interleukin-1β (IL-1β)	Fwd GCTACCTGTGTCTTTCCCGT Rev CATCTCGGAGCCTGTAGTGC	164
NM_007482.3	Arginase-1 (Arg1)	Fwd ACAAGACAGGGCTCCTTTCAG Rev TTAAAGCCACTGCCGTGTTC	105
NM_011434.2	Superoxidase dismutase (SOD1)	Fwd CAATGGTGGTCCATGAGAAACA Rev CCCAGCATTTCCAGTCTTTGTA	77
NM_001199177.1	Mitochondrial dynamin like GTPase (OPA1)	Fwd GCCCTTCTCTTGTTAGGTTCAC Rev ACACCTTCCTGTAATGCTTGTC	88
NM_007540.4	Brain derived neurotrophic factor (BDNF)	Fwd CGAGTGGGTCACAGCGGCAG Rev GCCCCTGCAGCCTTCCTTGG	160
NM_001310086.1	Activity-dependent neuroprotective protein (ADNP)	Fwd GTGACATTGGGTTGGAATACTGT Rev AGGTTTTGTCCGATAGTCCTGA	149
NM_016989.2	Pituitary adenylate cyclase-activating polypeptide (PACAP)	Fwd AGGCTTACGATCAGGACGGA Rev CTCCTGTCGGCTGGGTAGTA	121
NM_053991.1	Vasoactive intestinal peptide (VIP)	Fwd CCTGGCGATCCTGACACTCT Rev CTGCAGCCTGTCATCCAACC	100
NM_213557.1	18S ribosomal subunit (s18)	Fwd GGCGGAAAATAGCCTTCGCT Rev AGCCCTCTTGGTGAGGTCAA	101

**Table 3:** Antibodies used in Western blots and immunohistochemistry.

Antibody	Dilution	Source (Cat. #)
Tyrosine hydroxylase (TH)	1:200 (WB) 1:500 (IHC-P)	Abcam (ab112)
Dopamine transporter (DAT)	1:1000	Abcam (ab128848)
Glial fibrillary acidic protein (GFAP)	1:1000	Abcam (ab68428)
Ionized calcium binding protein (Iba1)	1:1000	Sigma Aldrich (SAB2702364)
Mitochondrial dynamin like GTPase (OPA1)	1:1000	GeneTex (GTX129917)
Superoxidase dismutase (SOD1)	1:1000	GeneTex (GTX100554)
Pituitary adenylate cyclase-activating polypeptide (PACAP)	1:1000	GeneTex (GTX37576)
Vasoactive intestinal peptide (VIP)	1:1000	GeneTex (GTX129461)
Glyceraldehyde-3-phosphate dehydrogenase (GAPDH)	1:1000	BioRad (VPA00187)
Goat anti-Rabbit IgG HRP	1:10,000	BioRad (STAR208P)
Goat anti-Mouse IgG (H + L)-HRP	1:10,000	BioRad (1706516)

WB, Western blot; IHC-P, immunohistochemistry on paraffin embedded tissue.

## Supplementary Materials

The following supporting information can be downloaded at: https://www.mdpi.com/article/10.3390/ijms23031845/s1.
