# Supplementary material for: The Anxiolytic Drug Buspirone Prevents Rotenone-Induced Toxicity in a Mouse Model of Parkinson’s Disease
Source: Int J Mol Sci. 2022 Feb 6;23(3):1845. doi: 10.3390/ijms23031845 (PMC8837189; doi:10.3390/ijms23031845)
Supplement: Supplementary file 1 [file ijms-23-01845-s001.zip › ijms-1572723-supplementary.pdf]

The anxiolytic drug buspirone prevents rotenone-induced toxicity in a mouse model of Parkinson's disease

Sarah Thomas Broome [1] and Alessandro Castorina [1,*]

[1] Laboratory of Cellular and Molecular Neuroscience (LCMN), School of Life Sciences, Faculty of Science, University of Technology Sydney, Sydney, NSW, 2007, Australia
* Correspondence: Alessandro.Castorina@uts.edu.au

Supplementary Figures (1-6) and Figure Legends

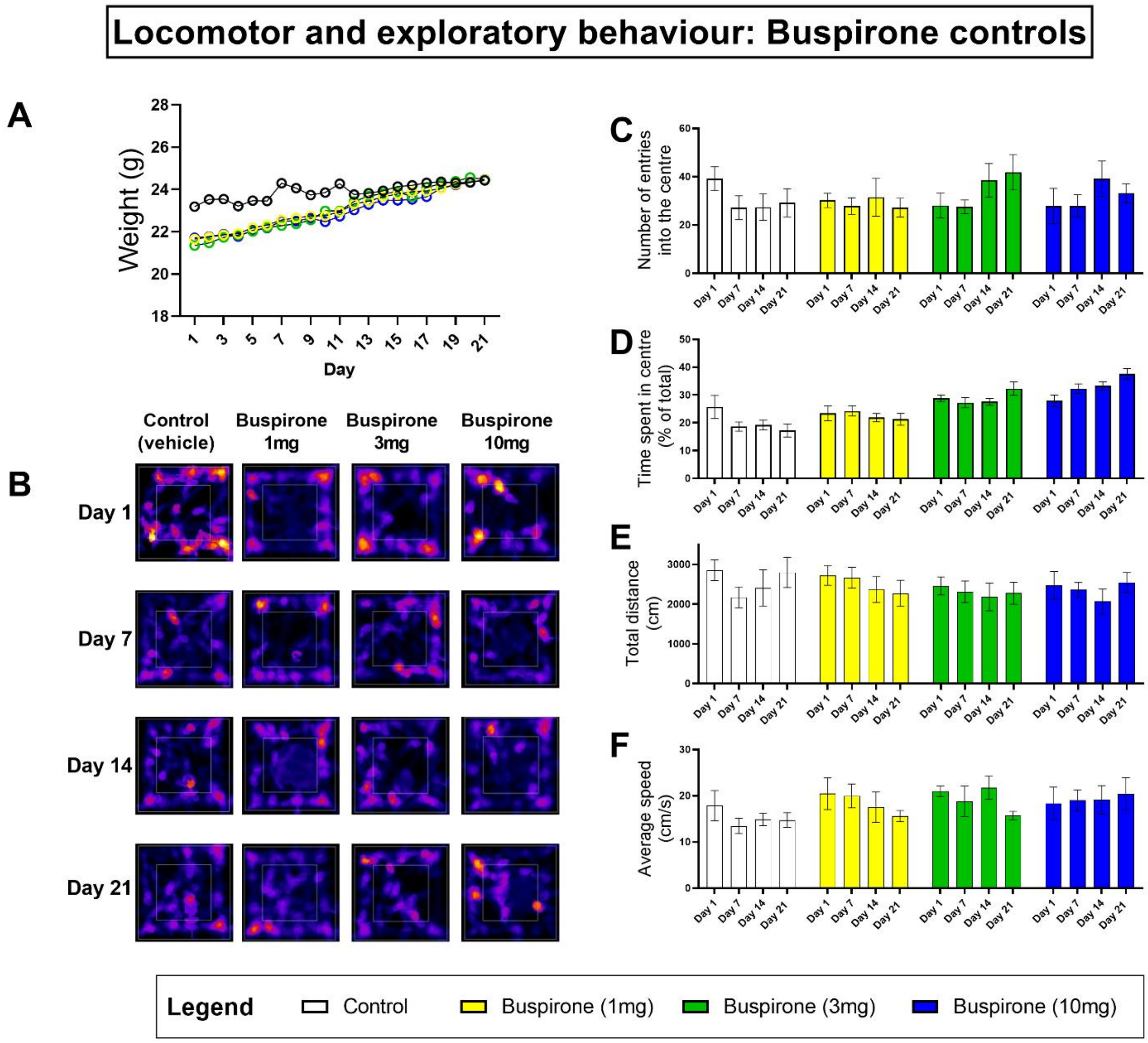

Supplementary Figure S1. Locomotor and exploratory behaviour of locomotor controls. Mice were weighed daily to monitor health and well-being (**A**). The open field (OF) test was used to assess for locomotor and exploratory behaviour in mice overtime. Representative heat maps generated from MouBeAt Software illustrating movement of mice during 5 min in OF (**B**). Exploratory behaviour was examined by the number of times (**C**) and the total time (**D**) a mouse spent in the entre quadrant (inner white square on heat maps). Locomotor activity was assessed by comparing the total distance travelled (**E**) and average speed (**F**). Comparisons were made within the same treatment group compared to baseline measurements. Data shown represents 7-12 mice per group. $p \leq 0.05$ was considered statistically significant, as determined by one-way ANOVA followed by Dunnett's post-hoc test.

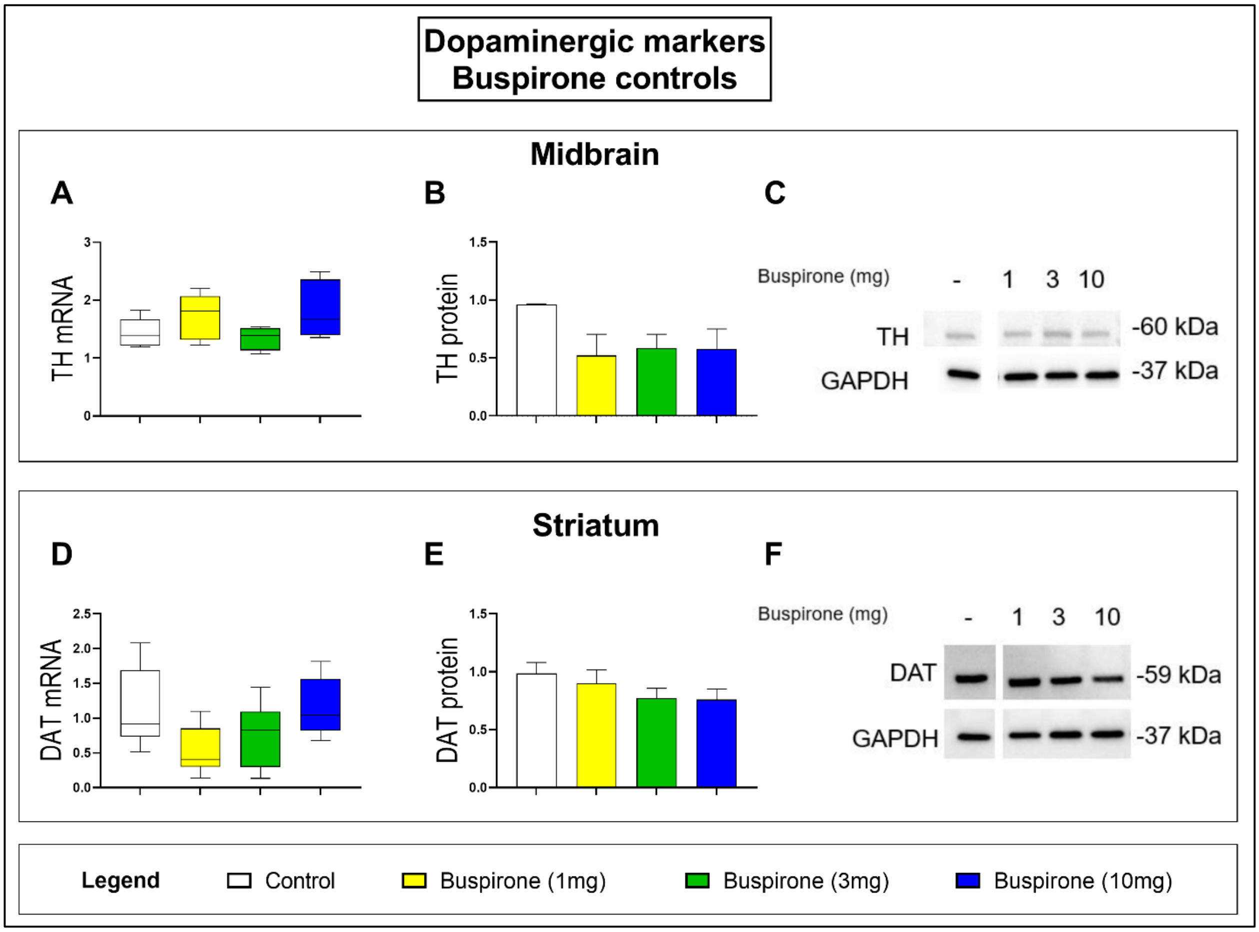

Supplementary Figure S2. Buspirone alone did not disturb the expression of nigro-striatal dopaminergic markers. TH mRNA (**A**) and protein expression (**B-C**) in the midbrain. Striatal DAT mRNA (**D**) and protein expression (**E-F**). Gene expression was measured by qRT-PCR and quantified using the $\Delta\Delta Ct$ method after normalization to s18 (the housekeeping gene). qRT-PCR results are reported as mean fold changes with respect to vehicle-treated control mice. Protein expression was determined by western blot and normalized to GAPDH (the loading control). Western blots show lanes removed from in text figure 2. Densitometry results presented as mean ± S.E.M. Data represents 3-4 mice per group. $p \leq 0.05$ was considered statistically significant, as determined by one-way ANOVA followed by Dunnett's post-hoc test. TH: tyrosine hydroxylase; DAT: dopamine transporter; GAPDH: glyceraldehyde 3-phosphate dehydrogenase; kDa: Kilodalton; s18: ribosomal protein s18 gene.

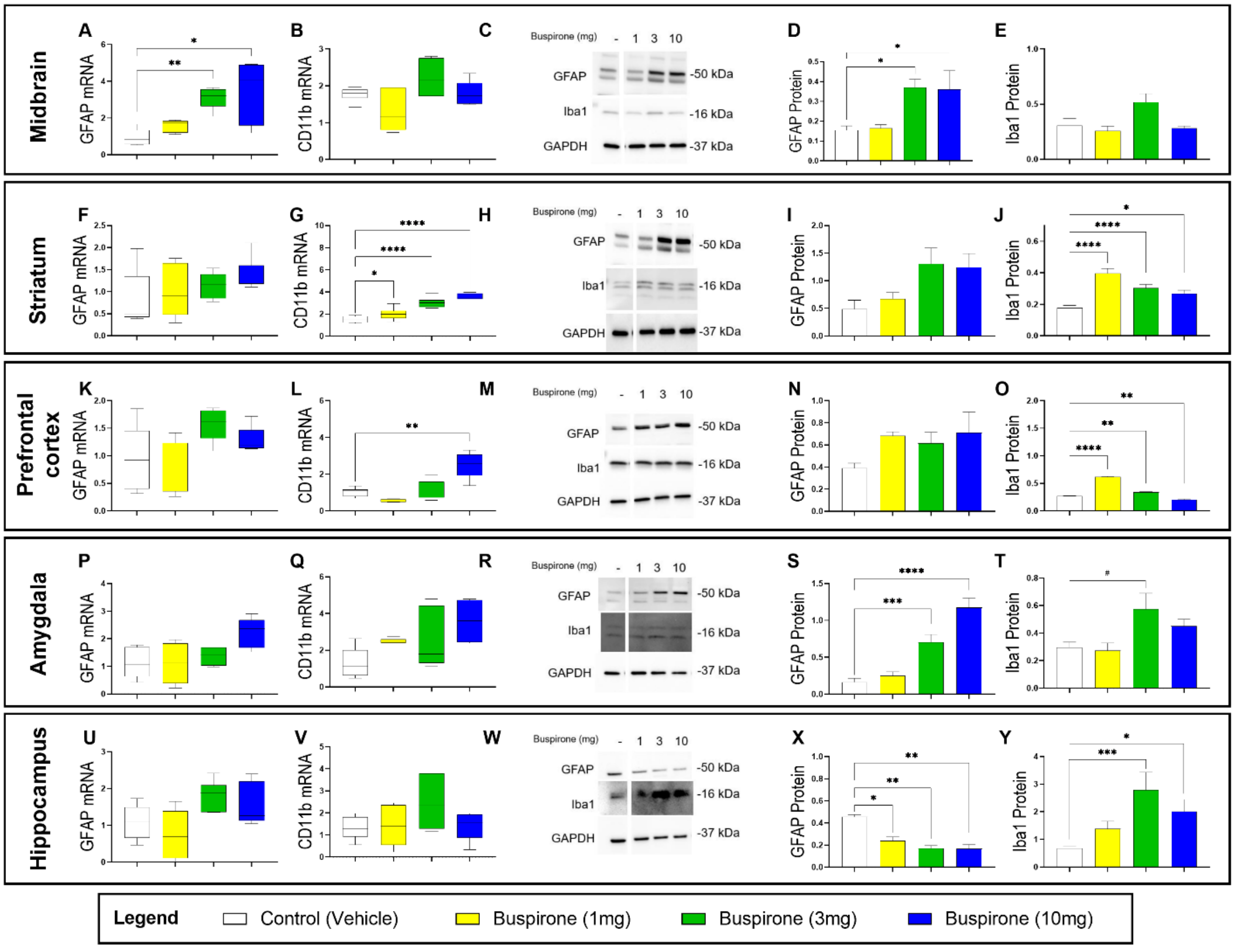

Supplementary Figure S3. Effect of buspirone treatment alone on the expression of glial cells markers. GFAP was used as an activation marker of astrocytes and CD11b and Iba1 were used collectively as markers of microglial activation. Real-time qPCR analyses of GFAP and CD11b in the midbrain (**A-B**), striatum (**F-G**), prefrontal cortex (**K-L**), amygdala (**P-Q**) and hippocampus (**U-V**), reported as mean fold changes calculated with the ΔΔCt method after normalization to s18 (the housekeeping gene). Western blot and densitometric analysis of GFAP and Iba1 in the midbrain (**C-E**), striatum (**H-J**), prefrontal cortex (**M-O**), amygdala (**R-T**) and hippocampus (**W-Y**). Densitometric results are expressed as mean ± S.E.M. Western blots show lanes removed from in text figure 3. All data represents 4-6 samples per treatment group. *$p < 0.05$, **$p < 0.01$, ***$p < 0.001$ or ****$p < 0.0001$ as determined by ANOVA followed by Dunnett's post-hoc test. GFAP: Glial Fibrillary Acidic Protein; Iba1: ionized calcium binding adapter molecule 1; s18: ribosomal protein s18 gene; GAPDH: Glyceraldehyde 3-phosphate Dehydrogenase; kDa: Kilodalton.

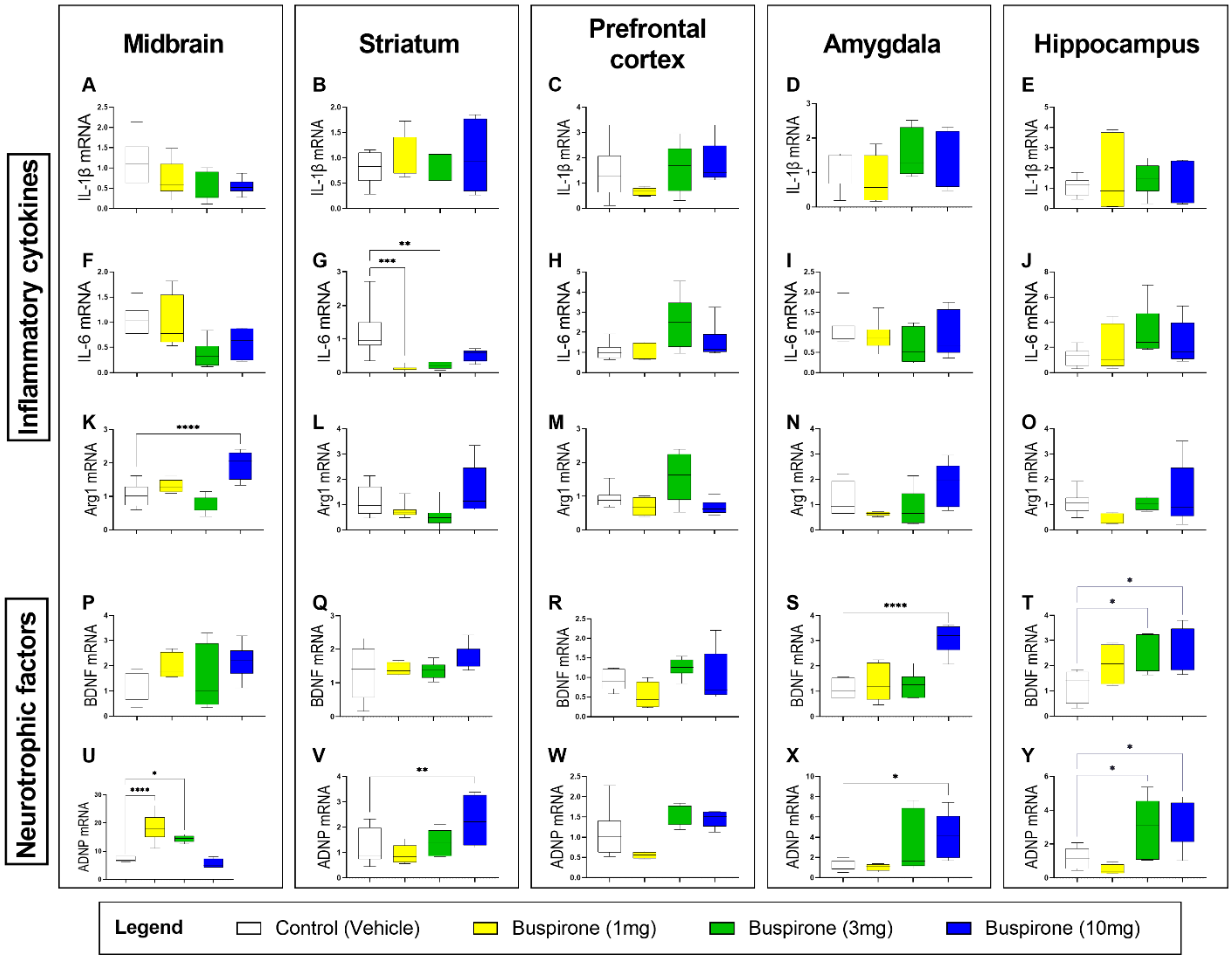

Supplementary Figure S4. Buspirone treatment alone, reduced basal levels of inflammatory cytokines and up-regulates the expression of neurotrophic factors in the brain. Real-time qPCR analyses of the pro-inflammatory cytokines IL-1β (**A-E**) and IL-6 (**F-J**), the anti-inflammatory cytokine Arg1 (**K-O**) and neurotrophic factors BDNF (**P-T**) and ADNP (**U-Y**) in the midbrain (box 1), striatum (box 2), prefrontal cortex (box 3), amygdala (box 4) and hippocampus (box 5), reported as mean fold changes calculated with the ΔΔCt method after normalization to s18 (the housekeeping gene. All data represents 5-8 samples per treatment group. *$p < 0.05$, **$p < 0.01$, ***$p < 0.001$ or ****$p < 0.0001$, compared to vehicle-treated controls, as determined by ANOVA followed by Dunnett's post-hoc test. IL-1β: interleukin-1beta: IL-6: interleukin-6; Arg1: arginase 1; BDNF: brain-derived neurotrophic factor; ADNP: activity-dependent neuroprotective protein; s18: ribosomal protein s18 gene; GAPDH: Glyceraldehyde 3-phosphate Dehydrogenase; kDa: Kilodalton.

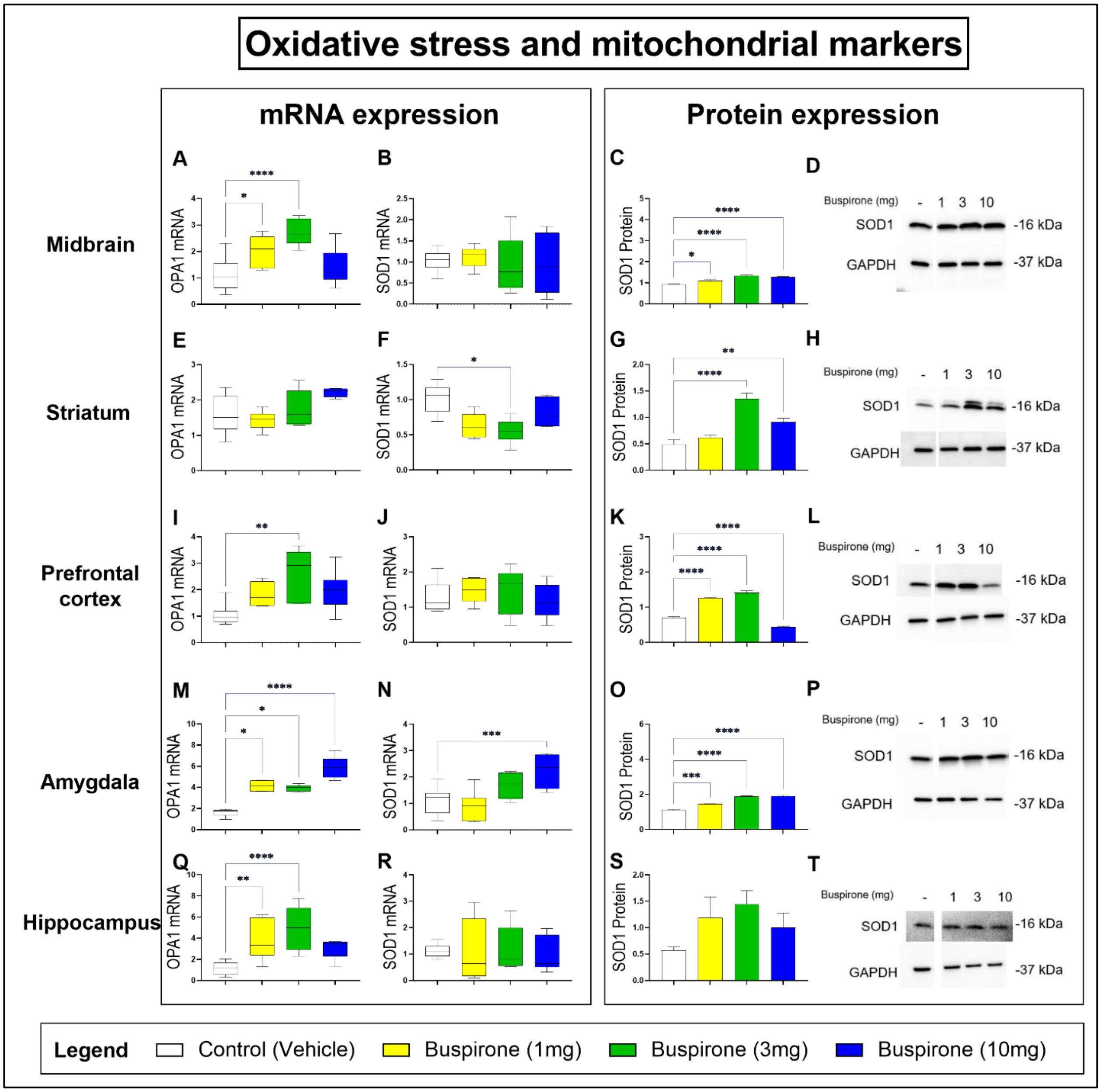

Supplementary Figure S5. Buspirone improves mitochondrial function and stimulates the expression of anti-oxidant enzymes. The mitochondrial marker, OPA1, was analysed to determine rotenone-induced mitochondrial damage and the antioxidant enzyme SOD1 was investigated as an indirect measure of oxidative stress. Real-time qPCR analyses of OPA1 and SOD1 mRNA expression in the midbrain (**A-B**), striatum (**E-F**), prefrontal cortex (**I-J**), amygdala (**M-N**) and hippocampus (**Q-R**) following administration of rotenone (10mg/kg) and/or buspirone (1, 3 or 10mg) daily for 21 days. Fold-changes were calculated using the ΔΔCt method after normalization to s18 (ribosomal protein s18 gene), the housekeeping gene. Each data point represents the mean value from n = 5-8 mice per each group. Representative Western blots and densitometry of SOD1 protein expression in the midbrain (**C-D**), striatum (**G-H**), prefrontal cortex (**K-L**), amygdala (**O-P**) and hippocampus (**S-T**). Protein expression was normalized to GAPDH, the loading control. Western blots show lanes removed from in text figure 5. Densitometric results are

expressed as mean ± S.E.M from n = 5-8 mice per each group. *p < 0.05, **p < 0.01, ***p < 0.001 or ****p < 0.0001, compared to vehicle-treated controls, as determined by ANOVA followed by Dunnett's post-hoc test. OPA1: Mitochondrial dynamin like GTPase; SOD1: superoxide dismutase 1; s18: ribosomal protein s18 gene; GAPDH: glyceraldehyde 3-phosphate dehydrogenase; kDa: Kilodalton.

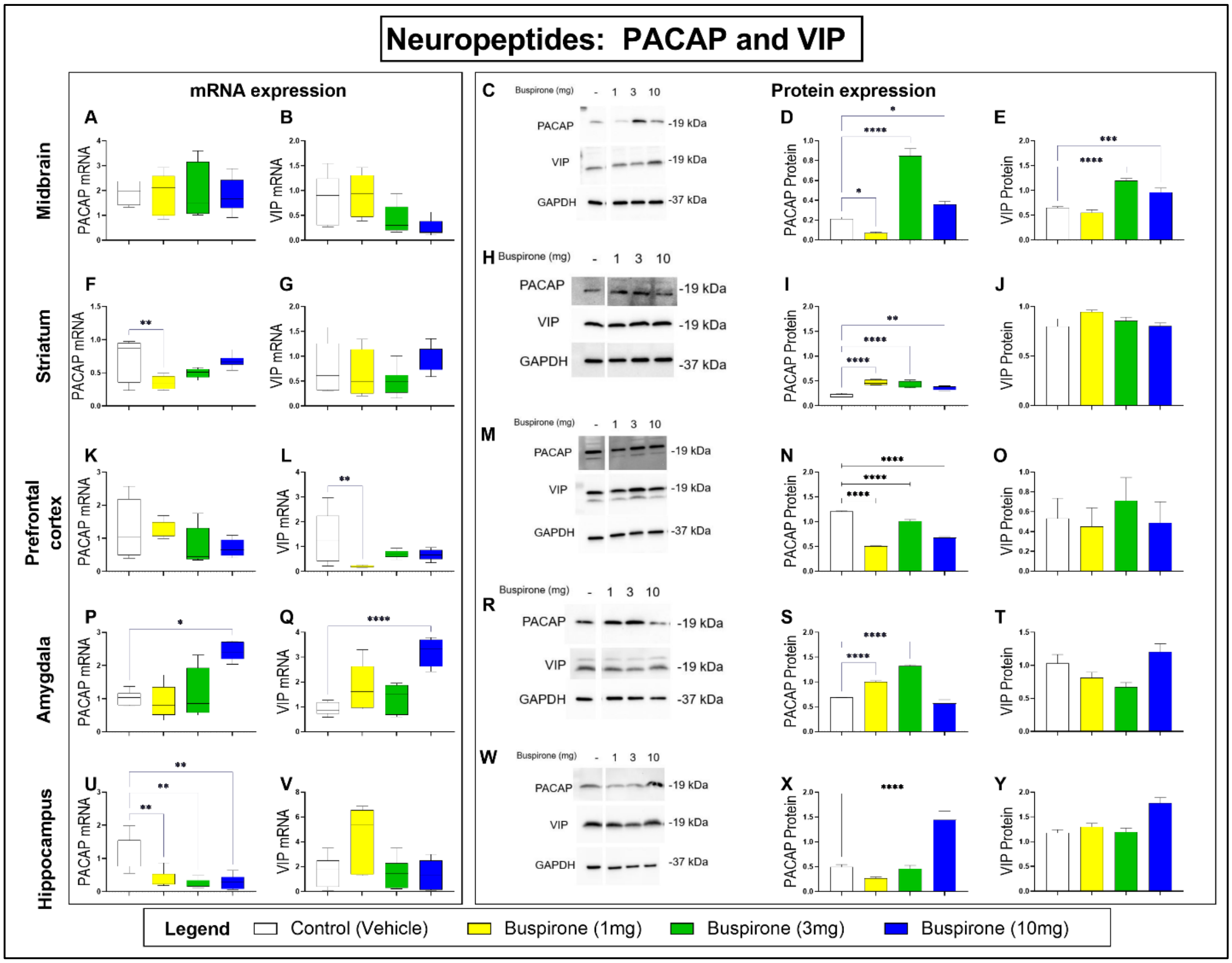

Supplementary Figure S6. Buspirone alters the endogenous expression of the neuropeptides PACAP and VIP. Real-time qPCR analyses of PACAP and VIP mRNA expression in the midbrain (**A-B**), striatum (**F-G**), prefrontal cortex (**K-L**), amygdala (**P-Q**) and hippocampus (**U-V**) following administration of rotenone (10mg/kg) and/or buspirone (1, 3 or 10mg) daily for 21 days. Fold-changes were calculated using the ΔΔCt method after normalization to s18 (ribosomal protein s18 gene), the housekeeping gene. Each data point represents the mean value from n = 5-8 mice per each group. Representative Western blots and densitometry of PACAP and VIP protein expression in the midbrain (**C-E**), striatum (**H**-J), prefrontal cortex (**M-O**), amygdala (**R-T**) and hippocampus (**W-Y**). Protein expression was normalized to GAPDH, the loading control. Western blots show lanes removed from in text figure 6. Densitometric results are expressed as mean ± S.E.M from n = 5-8 mice per each group. *p < 0.05, **p < 0.01, ***p < 0.001 or ****p < 0.0001, compared to vehicle-treated controls, as determined by ANOVA followed by Dunnett's

post-hoc test. PACAP: pituitary adenylate cyclase-activating peptide; VIP: vasoactive intestinal peptide; s18: ribosomal protein s18 gene; GAPDH: glyceraldehyde 3-phosphate dehydrogenase; kDa: Kilodalton.
